# High prevalence of vitamin D deficiency among normotensive and hypertensive pregnant women in Ghana

**DOI:** 10.1186/s12884-021-03802-9

**Published:** 2021-04-26

**Authors:** Linda Ahenkorah Fondjo, Worlanyo Tashie, William K. B. A. Owiredu, Enoch Appiah Adu-Gyamfi, Laila Seidu

**Affiliations:** 1 Department of Molecular Medicine, SMD, KNUST, Kumasi, Ghana; 2 Department of Physiology, University of Cape Coast, Cape Coast, Ghana; 3 Comboni Hospital, Ho, Volta Region Ghana

**Keywords:** Preeclampsia, Normotensive, Vitamin D, 25-hydroxyvitamin D, Dyslipidemia, Preterm, Ghana

## Abstract

**Background:**

Hypovitaminosis D in pregnancy is associated with adverse health outcomes in mothers, newborns and infants. This study assessed the levels of 25-hydroxyvitamin D [25(OH)D] in normotensive pregnancies and in preeclampsia, evaluated the association between vitamin D deficiency and preeclampsia risk; and determined the foeto-maternal outcome in preeclamptic women with vitamin D deficiency.

**Methods:**

This case-control study was conducted among pregnant women who visited the Comboni Hospital, in Ghana from January 2017 to May 2018 for antenatal care. A total of 180 pregnant women comprising 88 preeclamptic women (PE) and 92 healthy normotensive pregnant women (NP) were recruited. Socio-demographic, clinical and obstetric data were obtained using validated questionnaires. Blood pressure and anthropometrics were measured, and blood samples were collected for the estimation of 25- hydroxyvitamin D [25(OH)D] using enzyme-linked immunosorbent assay technique. Lipids (total cholesterol, triglycerides, HDL-cholesterol and LDL-cholesterol) were also estimated.

**Results:**

A total of 81.7% of the study participants had vitamin D deficiency. Of these, 88.6% of the women with PE had vitamin D deficiency compared to 75.0% in the NP. Vitamin D levels were significantly reduced in the PE women compared to the normotensive pregnant women (*p* = 0.001). A higher proportion of the preeclamptic women who were vitamin D deficient had preterm delivery (*p* < 0:0001) and delivered low birth weight infants (*p* < 0:0001), and infants with IUGR (*p* < 0:0001) compared to the control group (*p* < 0:0001). Pregnant women with PE presented with significant dyslipidemia, evidenced by significantly elevated TC (*p* = 0.008), LDL (*p* < 0.0001), triglycerides (*p* = 0.017) and a significantly reduced HDL (*p* = 0.001) as compared to NP. In the preeclamptic women, serum 25(OH) D showed an inverse, but not significant association with TC (β = − 0.043, *p* = 0.722, TG (β = − 0.144, *p* = 0.210) and LDL (β = − 0.076, *p* = 0.524) and a positive, but not significant association with HDL (β = 0.171, *p* = 0.156).

**Conclusion:**

The prevalence of vitamin D deficiency is high in both normotensive pregnancies and pregnancies complicated by preeclampsia but amplified in preeclampsia. Higher proportion of pregnant women with hypovitaminosis D had preterm babies and delivered low birth weight neonates. Additional studies are needed to explore the potential benefits and optimal dosing of vitamin D use in pregnancy, especially in sub-Saharan Africa.

**Supplementary Information:**

The online version contains supplementary material available at 10.1186/s12884-021-03802-9.

## Background

The etiology of preeclampsia still remains inconclusive, with many countries still grappling its devastating effects on both mother and child. Aside vitamin D being a seco-steroid that primarily regulates calcium, phosphate and bone metabolism in the body [1], it is also known to have anti-inflammatory, anti-atherogenic and immune boosting properties. Several existing evidences allude to the etiology and preventive role of vitamin D in preeclampsia [2–4]. Vitamin D can be obtained from the exposure of the skin to sunlight, from diet, fortified dietary products and nutritional supplements. It exists in two forms: vitamin D_3_, and vitamin D_2_ [5]. The D_3_ form is produced by animals, including humans. It is either obtained from endogenous synthesis when the skin is exposed to ultraviolet B (UV-B) radiation of the sun or from diet such as fatty fish or eggs [1, 4]; whereas vitamin D_2_ is produced by plants [6]. Vitamin D_3_ is acted upon by 25-hydroxylase in the liver to form 25-hydroxyvitamin D [25(OH)D]- the major circulating form of vitamin D; and this is used as the determinant of vitamin D status in the body [7, 8]. The 25(OH) D is further hydroxylated in the kidneys by 1-α hydroxylase (a cytochrome P450 enzyme) to 1α,25-dihydroxyvitamin D [1,25(OH)_2_D]. The 1,25(OH)_2_D is the active form of vitamin D that acts on vitamin D receptors throughout the body to exert its functions [9–11].

Vitamin D deficiency is considered a worldwide public health problem, with an estimated one billion people suffering from vitamin D insufficiency or deficiency [12]. Adequate maternal vitamin D level is important during pregnancy as both the pregnant woman and the growing fetus depend largely on maternal vitamin D stores [13]. Vitamin D is essential in embryogenesis, especially fetal skeletal development and calcium homeostasis [14–16]. Evidence, however, indicates that normal pregnancy presents with alterations in maternal vitamin D metabolism to support fetal development [15]. In spite of it being a modifiable risk factor, vitamin D deficiency during pregnancy increases the risks of maternal and fetal complications [2, 17]. Low vitamin D status during pregnancy has been linked to pregnancy complications such as preeclampsia, preterm birth and low birth weight babies [2, 3]. Although, hypovitaminosis D in pregnancy is associated with adverse health outcomes in mothers, newborns and infants, only few studies have addressed the influence that hypovitaminosis D has on preeclampsia risk in the Ghanaian setting where sunshine is considered to be in abundance. In this study, we assessed the prevalence of vitamin D deficiency in normotensive pregnancies and in preeclampsia, evaluated the association between vitamin D deficiency and risk of preeclampsia; and determined the foeto-maternal outcome in preeclamptic women with vitamin D deficiency. Our findings will contribute to understanding the relevance of early vitamin D screening and supplementation during pregnancy.

## Materials and methods

### Study design and study setting

This case-control study was conducted at the Comboni Hospital, from January 2017 to May 2018. The hospital is located at Sogakope in the South Tongu district of the Volta region of Ghana with a population of 87,950 (Ghana Statistical Services, 2010). Comboni Hospital has a 50-bed capacity and provides health services to clients from the Southern part of the Volta Region, most especially with referrals from the South Tongu district, North Tongu district, Keta Municipality and Ketu North district.

### Study participants

One hundred and eighty (180) pregnant women comprising 88 preeclamptic women and 92 healthy normotensive pregnant women with gestational age > 20 weeks were recruited into this study. The study participants were aged between 18 and 40 years. The preeclamptic women were recruited by a qualified Obstetrician/Gynaecologist using the National High Blood Pressure Education Program Working Group diagnostic criteria [18]. Preeclampsia was diagnosed based on the new onset of hypertension and proteinuria (≥1+ reading on dipstick) occurring after the 20th week of gestation in a previously normotensive and non-proteinuric woman. Pregnant women with blood pressure ≥ 160/110 mmHg and proteinuria (≥3+ reading on dipstick) were classified as having severe preeclampsia while pregnant women with systolic blood pressure (SBP) of 140–159 mmHg, diastolic blood pressure (DBP) of 90–109 mmHg and proteinuria (≥1+ reading on dipstick) were classified as having mild preeclampsia. The control group consisted of age and gestation-matched healthy normotensive pregnant women. A detailed socio-demographic, medical and obstetric data was obtained from each participant using a well-structured validated questionnaire.

### Eligibility criteria

All recruited participants were singleton pregnant women, aged between 18 and 40 years and with gestational age > 20 weeks. Pregnant women with a confirmed diagnosis of preeclampsia and healthy normotensive pregnant women were included. This study excluded pregnant women who failed to give written consent. Pregnant women with twin gestation or history of chronic hypertension, diabetes mellitus, kidney disease, gestational diabetes or cardiovascular disorders and microbial infections were also excluded.

### Blood pressure measurement

The blood pressure of each participant was measured in the morning prior to venous blood sample collection. Each participant was asked to sit down comfortably, extend the left arm on a table and then relax for 10 min. An automated Accoson mercury sphygmomanometer (Essex, United Kingdom) was used by trained personnel to measure the systolic blood pressure and diastolic blood pressure of each participant according to standard guidelines [19]. Blood pressure was measured twice for each participant, 6–15 min apart. The mean blood pressure of the two measurements was reported as the blood pressure of each participant.

### Determination of early gestation BMI

The weight and height of each participant measured during the first antenatal visit was obtained from the maternal record of each participant. The weight was measured in kilograms while height was measured in centimeters. The Body Mass Index (BMI) was calculated as the ratio of body weight in kilograms to height in square meter.

### Determination of infant birth weight and intrauterine growth restriction

All Infant birth weight and estimated fetal weight (EFW) were obtained from the maternal record of each participant. All infants were weighed at birth using a weighing scale (Docbel Braun Baby Classic Weighing Scale, New Delhi, India). Neonates who weighed < 2.5 Kg at birth were considered as having low birth weight whereas neonates who weighed ≥2.5 Kg at birth were classified as having normal weight. Using the smoothed percentiles of the EFW for gestational age obtained from ultrasound measurements, we classified IUGR using the 10th percentile (see Additional File 1 for smoothed percentiles of estimated fetal weight (grams) for gestational age).

### Blood sample collection and biochemical assay

For the biochemical assay, about eight milliliters (8 ml) of venous blood was drawn from each participant into serum separator test tubes. After clotting, the samples were centrifuged at 4000 rpm for 8 min. The serum was separated and stored at − 20 °C until assayed. The stored serum was used to estimate 25(OH) D and lipid profile [total cholesterol, triglycerides, high density lipoprotein (HDL)]. All blood samples were collected early in the morning after 8–12 h of overnight fast. 25(OH) D was estimated using an enzyme-linked immunosorbent assay (ELISA) technique. The optical density was read at 450 nm using Mindray MR-96A microplate reader (Shenzhen, China). The serum level of 25(OH) D in the test samples was calculated from the standard curve plotted using the different standards with known antigen concentration. Serum levels of 25(OH) D were stratified into severe deficiency, mild to moderate deficiency and optimal as follows: 10-19 ng/mL, 10-19 ng/mL and 20-50 ng/mL respectively [14, 20]. Total cholesterol, triglycerides and HDL were estimated enzymatically using an automated Mindray BS-120 Chemistry Analyzer (Shenzhen, China). The absorbance of each analyte was determined spectrophotometrically at wavelength of 505 nm. Serum concentration of low density lipoprotein (LDL) was calculated using Friedewald’s equation as follows:

$$ \mathrm{LDL}= Total\ cholesterol- HDL-\frac{Triglycerides}{2.2}\  mmol/L $$

### Statistical analysis

Data was analyzed using IBM SPSS (Version 25.0. Armonk, NY: IBM Corp). The dataset had no missing data. Continuous data were reported as mean ± standard deviation (SD), while categorical data were reported as proportions and percentages. Comparison of the continuous data was carried-out using the independent sample t-test. Chi square (*χ*^*2*^) and Fisher’s exact test were used to compare categorical data. After adjusting for confounders (maternal age, BMI, gestational age, marital status, contraceptive use and history of abortion); multivariate logistic regression analysis to determine the odds of vitamin D status in predicting preeclampsia and linear regression analysis to estimate the association between serum 25-hydroxy vitamin D and lipid profile parameters were carried out. Two-tailed tests were used for all analysis. Statistical significance level was considered at *p* < 0.05.

## Results

Table 1 summarizes the socio-demographic and obstetric variables of the preeclamptic women (PE) and the healthy normotensive pregnant women (NP). Fifty-one percent (51.1%) of the participants had basic education whereas 25.6 and 14.4% had completed secondary and tertiary education respectively. About 9 % (8.9%) of the study participants had no formal education. Both PE and NP presented with similar level of education (*p* = 0.660). A higher proportion of the study participants were married (76.7%). Whereas 74 % (73.9%) of the pregnant women were “Ewes”, 13.3 and 12.8% were “Akans” and “Gas” by ethnicity respectively. Majority of the study participants (62.8%) were self-employed. The PE and NP women differed in their marital statuses (*p* = 0.023) and family history of hypertension (*p* = 0.076). The difference in parity (*p* = 0.903) and gravidity (*p* = 0.158) between the PE and the NP women was insignificant. The percentage of women with history of abortion (*p* = 0.003) and contraceptive use (*p* < 0.0001) were significantly higher in the PE compared to the NP women (Table 1).

**Table 1:** Baseline characteristics of preeclamptic and normotensive pregnant women

Variables	Total (***n*** = 180)	NP (***n*** = 92)	PE (***n*** = 88)	***p-value***
**Highest of Level Educational**				0.660
No education	16 (8.9)	7 (7.6)	9 (10.2)	
Basic education	92 (51.1)	45 (48.9)	47 (53.4)	
Secondary education	46 (25.6)	27 (29.3)	19 (21.6)	
Tertiary education	26 (14.4)	13 (14.1)	13 (14.8)	
**Ethnicity**				0.116
Ewe	133 (73.9)	64 (69.6)	69 (78.4)	
Ga	23 (12.8)	11 (12.0)	12 (13.6)	
Akan	24 (13.3)	17 (18.5)	7 (8.0)	
**Marital Status**				0.023
Single	42 (23.3)	28 (30.4)	14 (84.1)	
Married	138 (76.7)	64 (69.6)	74 (15.9)	
**Occupation**				0.313
Unemployed	37 (20.6)	23 (25.0)	14 (15.9)	
Self-employed	113 (62.8)	54 (58.7)	59 (67.0)	
Civil servant	30 (16.7)	15 (16.3)	15 (17.0)	
**Parity**				0.903
Nulliparous	62 (34.4)	33 (35.9)	29 (33.0)	
Primiparous	49 (27.2)	25 (27.2)	24 (27.3)	
Multiparous	69 (38.3)	34 (37.0)	35 (39.8)	
**Gravidity**				0.158
Primigravida	58 (32.2)	33 (35.9)	25 (28.4)	
Secundigravida	43 (23.9)	25 (27.2)	18 (20.5)	
Multigravida	79 (43.9)	34 (37.0)	45 (51.1)	
**Family History of Hypertension**				0.076
Yes	12 (6.7)	3 (3.3)	9 (10.2)	
No	168 (93.3)	89 (96.7)	79 (89.8)	
**History of Abortion**				0.003
Yes	31 (17.2)	8 (8.7)	23 (26.1)	
No	149 (82.8)	84 (91.3)	65 (73.9)	
**Contraceptive use**				
Yes	57 (31.7)	18 (19.6)	39 (44.3)	< 0.0001
No		74 (80.4)	49 (55.7)	

Values are presented as frequency (proportion). Variables in PE were compared to NP using χ^2^ or Fischer’s exact test (when *n* < 5). Statistically significant level was given at *p*-value < 0.05

*PE* Preeclamptic women, *NP* Normotensive pregnant control

The anthropometric characteristics of PE and NP are shown in Table 2. PE is associated with significantly increased pre-gestational weight (*p* = 0.001), pre-gestational BMI (*p* = 0.023) and BMI at sampling (*p* = 0.008) compared to NP (Table 2).

**Table 2:** Anthropometric characteristics of the preeclamptic and normotensive pregnant women

Parameters	NP (***n*** = 92)	PE (***n*** = 88)	***p-value***
Height (m)	1.57 ± 0.09	1.60 ± 0.08	0.030
Pre-gestational weight (kg)	65.71 ± 13.84	73.01 ± 14.90	0.001
Pre-gestational BMI (kg/m^2^)	26.81 ± 5.47	28.80 ± 6.16	0.023
Weight at sampling (kg)	70.40 ± 14.32	78.94 ± 15.48	< 0.0001
BMI at sampling (kg/m^2^)	28.73 ± 5.64	31.15 ± 6.46	0.008
% body weight gain at sampling	7.37 ± 3.56	8.35 ± 3.76	0.073

Data are presented as mean ± SD. The means of the clinical variables in PE were compared with those of NP using independent (unpaired) t-test. Statistically significant level was set at *p*-value < 0.05

*PE* Preeclamptic women, *NP* Normotensive pregnant control, *BMI* Body mass index

The clinical variables of the study participants are summarized in Table 3. Both the PE and the NP women presented with similar maternal ages (*p* = 0.462) and gestational age at sampling (*p* = 0.104). However, the PE women presented with significant dyslipidemia, evidenced by significantly elevated TC (*p* = 0.008), LDL (*p* < 0.0001), triglycerides (*p* = 0.017) and a significantly reduced HDL (*p* = 0.001) on comparison to NP. The PE women had significantly reduced 25(OH) D levels compared to NP (*p* = 0.001) (Table 3).

**Table 3:** Clinical and biochemical variables of the study participants

Parameters	Total (***n*** = 180)	NP (***n*** = 92)	PE (***n*** = 88)	***p-value***
Age (years)	29.00 ± 4.84	28.74 ± 4.95	29.27 ± 4.74	0.462
GA at sampling (weeks)	31.24 ± 3.29	30.85 ± 3.08	31.65 ± 3.48	0.104
TC (mmol/L)	4.56 ± 1.26	4.32 ± 1.30	4.82 ± 1.18	0.008
TG (mmol/L)	1.14 ± 0.57	1.04 ± 0.44	1.24 ± 0.66	0.017
LDL (mmol/L)	2.90 ± 1.18	2.63 ± 1.05	3.19 ± 1.24	< 0.0001
HDL (mmol/L)	1.14 ± 0.50	1.23 ± 0.45	1.04 ± 0.54	0.001
Mean 25(OH) D (ng/mL)	14.32 ± 7.19	16.00 ± 8.14	12.56 ± 5.56	0.001

Data are presented as mean ± SD. The means of the clinical variables in PE were compared with NP using independent t-test. Statistically significant difference was considered at *p*-value < 0.05

*PE* Preeclamptic women, *NP* Normotensive pregnant control, *GA* Gestational age

The 25(OH) D status in normal pregnancy and preeclampsia are shown in Table 4. The overall prevalence of vitamin D deficiency in the study participants was 81.7% (147/180). Of these, 88.6% (78/88) of the PE women had vitamin D deficiency whereas 75.0% (69/92) of the NP women had vitamin D deficiency. The logistic regression analysis shows that participants with severe 25(OH) D deficiency were at about six-fold [aOR = 5.9, 95% CI (2.14 to 16.40), *p* = 0.001] increase in the odds of developing preeclampsia (Table 4).

**Table 4:** Prevalence of 25-hydroxyvitamin D status among the study participants after adjusting for confounders

25(OH) D status	NP (***n*** = 92)	PE (***n*** = 88)	***χ***^***2***^, df, (***p-value***)	aOR (95% CI)	***p-value***
Severe deficiency	32 (34.8%)	51 (58.0%)	10.95, 2, (0.004)	5.92 (2.14–16.40)	**0.001**
Mild to moderate deficiency	37 (40.2%)	27 (30.7%)		2.13 (0.76–5.95)	0.151
Optimal level	23 (25%)	10 (11.4%)		1.0 (reference)	

Statistically significant level was set at *p*-value < 0.05

*PE* Preeclamptic women, *NP* Normotensive pregnant control, *χ*^*2*^*, df* Chi-square, degree of freedom, *aOR* adjusted odds ratio, *CI* Confidence interval, *25 (OH)D* 25-hydroxyvitamin D. *PE* Preeclamptic women, *NP* Normotensive pregnant control

Table 5 summarizes the association between 25(OH) D and the lipid profile parameters in pregnant women with vitamin D deficiency (severe deficiency and mild to moderate deficiency) after adjusting for maternal age, BMI, gestational age, marital status, contraceptive use and history of abortion. In the preeclamptic women, serum 25(OH) D showed an inverse, but not significant association with TC (β = − 0.043, *p* = 0.722), TG (β = − 0.144, *p* = 0.210) and LDL (β = − 0.076, *p* = 0.524) and a positive, but not significant association with HDL (β = 0.171, *p* = 0.156) (Table 5).

**Table 5:** The association between serum 25(OH) D concentration and serum lipids in pregnant women with vitamin D deficiency after adjusting for confounders

Variables	Normal pregnancy (***n*** = 69)		Preeclampsia (***n*** = 78)	
	β coefficient	***p-value***	β coefficient	***p-value***
TC (mmol/L)	0.266	0.041	−0.043	0.722
TG (mmol/L)	0.169	0.200	−0.144	0.210
LDL (mmol/L)	0.201	0.134	−0.076	0.524
HDL (mmol/L)	0.161	0.227	0.171	0.156

Statistically significant level was set at *p*-value < 0.05

*TC* Total cholesterol, *TG* Triglycerides, *LDL* low density lipoprotein, *HDL* high density lipoprotein, *25 (OH)D* 25-hydroxyvitamin D

Table 6 summarizes the pregnancy characteristics and birth outcomes in relation to vitamin D status in the study participants. A higher proportion of the preeclamptic women who were vitamin D deficient had preterm delivery (*p* < 0:0001) and delivered low birth weight infants (*p* < 0:0001), and infants with IUGR (*p* < 0:0001) compared to the control group (*p* < 0:0001) (Table 6).

**Table 6:** Pregnancy characteristics and birth outcomes of the study participants in relation to 25-hydroxyvitamin D status

Variables	Normal pregnancy (***n*** = 92)		Preeclampsia (***n*** = 88)		***p-value***
	Vitamin D Status [n (%)]				
	Deficient	Sufficient	Deficient	Sufficient	
**Infant birth weight**					< 0.0001
Normal	61 (66.3)	22 (23.9)	34 (38.6)	7 (8.0)	
Low	8 (8.7)	1 (1.1)	44 (50.0)	3 (3.4)	
**IUGR**					< 0.0001
Yes	69 (75.0)	23 (25.0)	30 (34.1)	3 (3.4)	
No	0 (0.0)	0 (0.0)	48 (54.5)	7 (8.0)	
**Status of delivery**					< 0.0001
Term	66 (71.7)	23 (35.0)	38 (43.2)	7 (8.0)	
Preterm	3 (3.3)	0 (0.0)	40 (45.5)	3 (3.4)	

χ^2^ or Fischer’s exact test (when *n* < 5) was used to compare the categorical data. Statistically significant level was set at *p*-value < 0.05

*IUGR* Intrauterine growth restriction

## Discussion

In this study, we assessed the prevalence of vitamin D deficiency in normotensive pregnant women and preeclamptic women in Ghana. The overall prevalence of vitamin D deficiency among the entire study participants was 81.7%. The prevalence in the preeclamptic women was 88.6%, whereas the normotensive pregnant women had a prevalence of 75.0%.

The high prevalence of vitamin D deficiency observed in the pregnant women in this study is similar to the observations made in other studies [3, 17, 21]. In a cross-sectional study among Saudi Arabian pregnant women, Al-Faris et al.*,* observed a prevalence of over 90% [3]. Choi et al.*,* [17] also reported the prevalence of vitamin D deficiency in pregnant Korean women to be 77.3%. In one retrospective Chinese study by Song and co-workers [13], a prevalence of 90% vitamin D deficiency was reported. On the contrary, Bärebring et al., [22] revealed the prevalence of vitamin D deficiency among pregnant women in Sweden as 10% in the entire population; 2% among women born in North Europe; and 50% among women born in Africa. Hashemipour and colleagues [23], however, observed no difference in vitamin D levels between preeclamptic women and the normotensive pregnant women in Iran. The differences in the findings of these studies could be attributed to the lack of uniformity in the cut-offs used to define vitamin D deficiency in the various studies, seasonal changes, diet, race, geographical location and use of vitamin D supplements [24]. Although pregnant women in Ghana are prescribed the daily recommended allowance of folic acid and other hematinic medicines, the use and prescription of vitamin D supplements is not a common practice, especially for pregnant women. It has for a long time been thought that the abundance of sunshine in the tropics provides adequate vitamin D. However, our study provides evidence that among both normotensive and preeclamptic women, vitamin D adequacy maybe a health problem worth giving attention to by policymakers.

Although, several factors affect vitamin D synthesis in the body [8, 9, 14], the skin is thought to represent the major source of vitamin D especially in the tropics. The observed high vitamin D deficiency in the present study suggests that vitamin D status might not mainly be dependent on exposure of the skin to sunlight; other factors such as diet, nutritional supplements and underlying health conditions may play essential roles, especially in the sub-Saharan African population where sunshine is in abundance. We have earlier reported a high prevalence of vitamin D deficiency among diabetics and among pre- and post-menopausal women [25, 26]. This present study accentuates the high prevalence of vitamin D deficiency not only among pregnant women but likely among the general populace. In this study, we report 6-fold increased odds of developing preeclampsia in women with severe vitamin D deficiency. This finding corroborates that of Mirzakhani and co-workers, who in their study identified that low vitamin D status in early pregnancy and throughout the pregnancy was associated with a higher risk of preeclampsia [27]. Early vitamin D screening and supplementation during pregnancy has been largely reported to be beneficial in reducing adverse pregnancy outcomes and complications [28]. Thus, a well-defined and monitored vitamin D supplementation regimen during pregnancy could be effective in reducing hypovitaminosis D in pregnancy and prevent preeclampsia in sub-Saharan Africa.

Also, our study demonstrates that higher proportion of the preeclamptic women with vitamin D deficiency presented with low-birth-weight neonates, IUGR and preterm delivery than women with vitamin D sufficiency. Vitamin D interacts with calcium and parathyroid hormone to influence fetal growth and development [29]. Some studies have reported a significant association between low-birth-weight neonates and maternal vitamin D deficiency [29, 30]. Reports indicate that about 15 million infants worldwide are born preterm [31]. Oluwole and co-workers demonstrated that pregnant women with vitamin D deficiency have an approximately 9-fold higher likelihood of preterm delivery [32]. Similarly, Kalok [33] observed a significant correlation between vitamin D level and gestational age at delivery. While, Rostami et al., found that vitamin D supplementation could reduce the risk of preterm delivery by about 40% [28], this evidence supports the notion that optimal vitamin D levels during pregnancy reduces adverse neonatal outcomes.

A healthy pregnancy is associated with physiologic hyperlipidemia to satisfy the demands of the developing fetus [34, 35]. Hyperlipidemia in normal pregnancy is non-atherogenic, and attributed to hormonal changes. However, in preeclampsia, there is dysregulation of lipid metabolism, which manifests as abnormal maternal serum lipid levels [34–36]. Consistent with this study, several other studies have reported dyslipidemia in preeclampsia and stated that abnormal lipid levels in pregnancy significantly contribute to the development of preeclampsia through the excessive deposition of triglycerides in the uterine spiral arteries which may result in endothelial dysfunction through the production of small dense LDL [34–38]. Although the women with preeclampsia in this study presented with abnormal serum lipid, no significant association between vitamin D deficiency and lipid was observed in both the normotensive women and preeclamptic women after adjusting for possible confounders. This may suggest that in this category of women, vitamin D status may not influence atherogenic risk. Other studies however, [39, 40] report significant association between vitamin D deficiency and lipids. It is noteworthy that in these studies, samples were collected in the first trimester and among normotensive pregnant women.

The present study was conducted only in the southern part of Ghana. Also, the vitamin D status of the participants were measured at the same time as the preeclampsia status. Further longitudinal/prospective studies using a larger sample size to explore the cause-effect relationship between vitamin D status and preeclampsia is necessary. Also, dietary recall was not possible in this study.

## Conclusion

Vitamin D deficiency is highly prevalent in both normal pregnancy and pregnancies complicated by preeclampsia. Higher proportion of pregnant women with hypovitaminosis D presented with adverse pregnancy outcomes. Pregnant women with preeclampsia presented with significant dyslipidemia. Further studies are needed to explore the potential benefits and optimal dosing of vitamin D use in pregnant women especially in sub-Saharan Africa. Investigating the mechanistic bases of the association between maternal hypovitaminosis D and adverse pregnancy outcomes should be considered in prospective studies.

## Supplementary Information

**Additional file 1.**

## Data Availability

The datasets used and/or analyzed during the current study are available from the corresponding author on reasonable request.
