# Supplementary material for: High prevalence of vitamin D deficiency among normotensive and hypertensive pregnant women in Ghana
Source: BMC Pregnancy Childbirth. 2021 Apr 26;21:331. doi: 10.1186/s12884-021-03802-9 (PMC8077698; doi:10.1186/s12884-021-03802-9)
Supplement: Supplementary file 1 — Additional file 1. [file 12884_2021_3802_MOESM1_ESM.zip › Smoothed percentiles of estimated birth weight- Amended.docx]

**High Prevalence of Vitamin D deficiency among normotensive and hypertensive pregnant women in Ghana**

Linda Ahenkorah Fondjo^1*^ Worlanyo Tashie^1^, William K. B. A. Owiredu^1^, Enoch Appiah Adu-Gyamfi^2^, Laila Seidu^3^

*^1^Department of Molecular Medicine, SMD, KNUST Ghana,^2^Department of Physiology, University of Cape Coast, Ghana, ^3^Comboni Hospital, Ho, Volta Region.*

**Corresponding Author*

[*linda.ahenkorahfondjo@yahoo.com*](mailto:linda.ahenkorahfondjo@yahoo.com)

*Department of Molecular Medicine, KNUST-Ghana*

*0000-0003-0252-3190*

**Smoothed percentiles of estimated birth weight (grams) for gestational age**


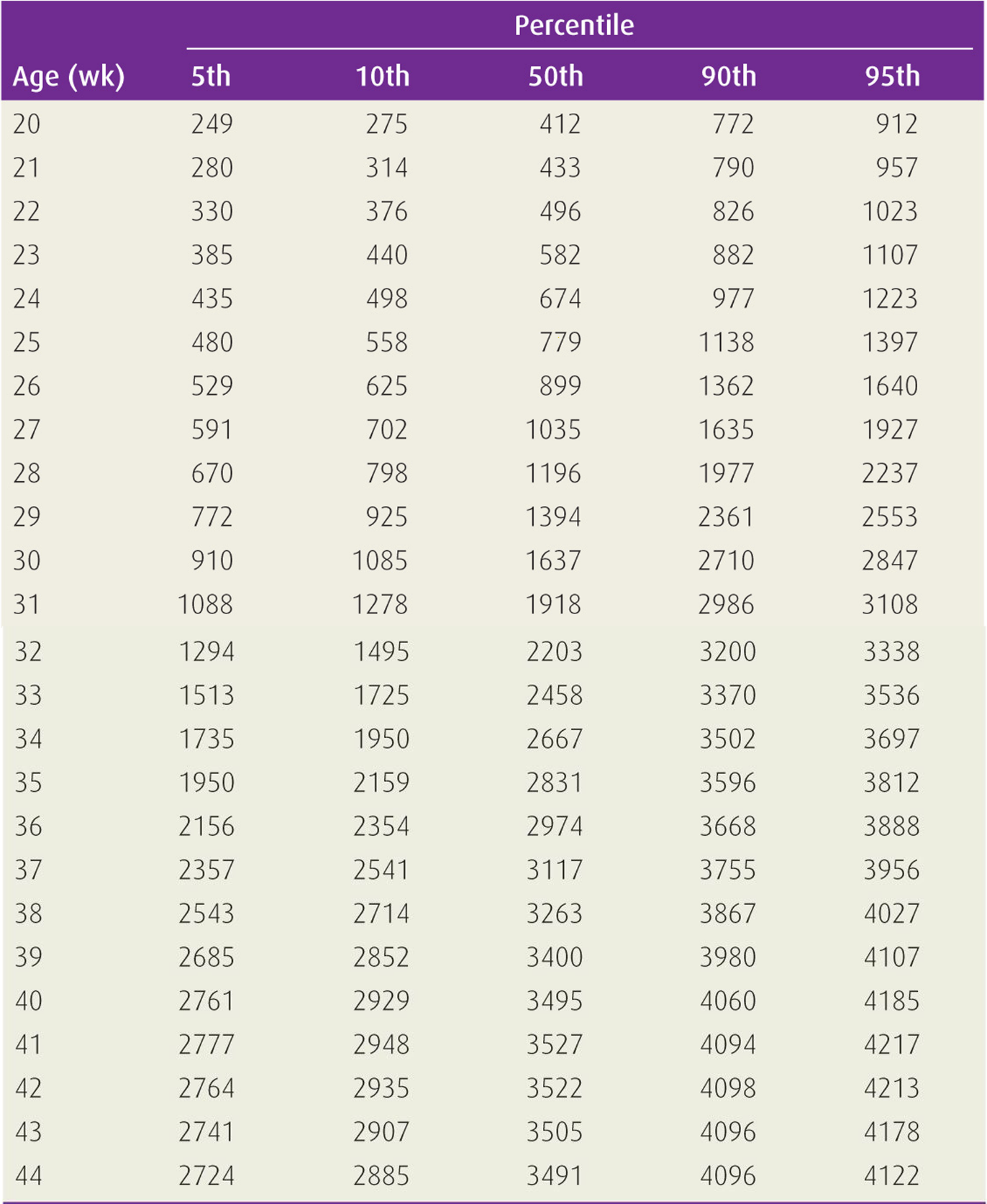


***Source***: Alexander GR, Himes JH, Kaufman RB, Mor J, Kogan M. A United States national reference for fetal growth. Obstetrics and gynecology. 1996;87(2):163-8 ([49](#_ENREF_49)).
